# Age-Stage, Two-Sex Life Table of *Atractomorpha lata* (Orthoptera: Pyrgomorphidae) at Different Temperatures

**DOI:** 10.3390/insects15070493

**Published:** 2024-07-01

**Authors:** Wenbo Li, Nawaz Haider Bashir, Muhammad Naeem, Ruilin Tian, Xinyue Tian, Huanhuan Chen

**Affiliations:** 1 College of Biological Resource and Food Engineering, Qujing Normal University, Qujing 655011, China; lwbnicklwb@163.com (W.L.); nawazhaider@caf.ac.cn (N.H.B.); naeem1633@yahoo.com (M.N.); 2 Key Laboratory of Biodiversity Conservationand Sustainable Utilization for College and University of Inner Mongolia Autonomous Region, College of Life Science and Technology, Inner Mongolia Normal University, Hohhot 010022, China; 3 School of Grassland Science, Beijing Forestry University, Beijing 100091, China; txy18943655384@163.com

**Keywords:** *Atractomorpha lata*, temperature, developmental period, effective accumulative temperature, fecundity, life table

## Abstract

**Simple Summary:**

*Atractomorpha lata* Motschoulsky (Orthoptera: Pyrgomorphidae) is a widespread phytophagous pest primarily found in the economic crop fields of Central Asian and Southeast Asian countries. Temperature plays a crucial role in determining its population growth and reproduction, and it is necessary to conduct in-depth research. We examined the developmental period of each stage and the number of eggs laid by female adults from the Yunnan Qujing population under different temperatures (16 to 32 °C). We calculated the developmental threshold temperature and effective accumulated temperature and constructed an age-stage-specific life table. The net reproduction rate (*R*_0_), total reproduction rate (*GRR*), and population trend index (*I*) were highest at 24 °C, which we identified as the optimal environmental temperature for the growth and reproduction of the Qujing population. Both high and low temperatures are not conducive to the survival and reproduction of its population. The results provide a scientific basis for predicting the potential distribution range, occurrence patterns in different regions, and field control strategies for this pest.

**Abstract:**

*Atractomorpha lata* Motschoulsky (Orthoptera: Pyrgomorphidae) has recently emerged as an important agricultural pest in China. Understanding the impact of temperature on its developmental period is crucial for predicting its population dynamics. This study systematically observed the biological characteristics of *A. lata* at five temperatures (16, 20, 24, 28, and 32 °C) using the age-stage, two-sex life table method. The effects of temperature on the developmental period, survival rate, and fecundity of *A. lata* were studied using fresh bean leaves as host. The results demonstrated that as temperature increased from 16 °C to 32 °C, the developmental period, preadult time, adult longevity, adult preoviposition period (APOP), and total preoviposition period (TPOP) significantly decreased. The developmental threshold temperatures for various stages were calculated, ranging from 10.47 °C to 13.01 °C, using the linear optimal method. As temperature increased, both the intrinsic rate of increase (*r*) and the finite rate of increase (*λ*) also increased, while the mean generation time (*T*) decreased. The optimal values of the net reproductive rate (*R*_0_ = 54.26 offspring), gross reproductive rate (*GRR* = 185.53 ± 16.94 offspring), and fecundity (169.56 ± 9.93 eggs) were observed at 24 °C. Similarly, the population trend index (*I*) for *A. lata* peaked at 24 °C (61.64). Our findings indicate that *A. lata* exhibits its highest population growth rate at 24 °C, providing a scientific basis for predicting its population dynamics in the field.

## 1. Introduction

*Atractomorpha lata* Motschoulsky belongs to the Pyrgomorphidae family (Orthoptera, Acridoidea) and is a common leaf-eating pest in the economic crop fields of Central and Southeast Asian countries. It is mostly found in agricultural, forestry, and pastoral areas of Mongolia, Russia, North Korea, South Korea, Japan, etc. [1]. In China, it is distributed in the Northeast Plain, the North China Plain, the middle and lower reaches of the Yangtze River Plain, and the Yunnan-Guizhou Plateau, including Inner Mongolia, Jilin, Liaoning, Hebei, Shandong, Shanxi, Shaanxi, Henan, Hubei, Hunan, Anhui, Shanghai, Guangdong, Guangxi, Shaanxi, Guizhou, and Yunnan [2,3,4]. In recent years, the occurrence of *Atractomorpha* harmful locusts in soybean production areas in southern China has increased, and it has become an important leaf-eating pest due to the mixed growth of other destructive locust species, resulting in heavy yield reduction in economic crops. The pest has a wide range of feeding habits, and the host plant species number as many as hundreds. The detrimental impact of this pest extends to a wide range of crops, including rice, soybean, corn, cotton, cabbage, cowpea, rape, sweet potato, sunflower, peanut, and citrus. Furthermore, its feeding habits resemble those of other detrimental grasshopper species, which can easily cause serious harm and damage to the agriculture, forestry, and animal husbandry economies [4,5,6]. *Atractomorpha lata* lay eggs and overwinter in soybean field soil. It feeds on soybean leaves from the seedling stage to the mature stage, and the damage period is long, seriously affecting soybean yield and quality [7]. There are few studies on the biological aspects of *A. lata*. In Hebei Province, Tian et al. investigated the distribution of harmful locusts in farmland ecosystems and identified *Atractomorpha lata*, *Atractomorpha sinensis*, *Atractomorpha sagittaris*, and *Atractomorpha heteroptera* [8]. Seven species of Acridoidea have been recorded in southern China’s economic crop planting areas, with *A. lata* being the most dangerous [9]. According to Liu et al., the overwintering eggs of the *A. lata* indoor population had a freezing point of −19.44 ± 0.40 °C and a supercooling point of −25.09 ± 0.18 °C [5]. Li et al. estimated that the eggs of the harmful locust Qujing population have a 11.44 °C threshold value of temperature of developmental stage and an effective cumulative temperature of 391.16 degree-days at room temperature [10]. Currently, there is lack of systematic study available on the effect of temperature on the growth, development, and reproduction of the *A. lata* population.

Insects are poikilothermic, and environmental temperature regulates insect growth, development, and determines their geographical distribution [6,11,12]. Within a suitable temperature range, insects have a consistent growth rate and a high rate of population survival [13]. *Atractomorpha sinensis* can complete the life cycle in the range of 16 °C to 32 °C. At 24 °C, the population survival rate is high, the fecundity is high, and the life cycle is short [6]. To determine the threshold temperature of developmental stage of insect, the effective accumulated temperature method is a valuable tool which is helpful for pest control and resource insect protection [10]. The traditional life table obtains the survival rate, fecundity, and life table parameters through the average development period of different developmental stages of insects [13]. It only considers the life process of female insects and represents the whole population with twice the number of female insects, ignoring the difference between female and male insects and the contribution of male insects to population growth [6]. Compared with the traditional age-specific life table of females, the age-stage two-sex life table is an important method to study the number of insect populations, evaluate pest control measures, formulate pest prediction schemes, and implement scientific pest control [14,15]. It can accurately analyze and understand the influence of external environmental conditions on biological growth and development, and help to obtain detailed life parameters under various conditions [16,17]. Our group used the age-stage bisexual life table to systematically study the growth, development, and reproduction of *Atractomorpha sinensis*, *Riptortus pedestris*, *Pectinophora gossypiella*, and other pests [6,13,18].

In the past century, the outbreak of locust plague in China mostly occurred in warm climate years [19]. *A. lata* has a wide distribution, a variety of habitat types, and a large geographical latitude and altitude range. For a long time, the agricultural and forestry plant protection departments in various places lacked monitoring of the dynamic occurrence and damage of harmful locust groups, resulting in an increase in the frequency of spring soybean planting areas in Northeast China and North China in recent years, spreading southward year by year, and the harm has been aggravated. Since the invasion of China in 1983, *Atractomorpha lata* has spread to vast areas except Xinjiang, Tibet, Qinghai, Gansu, Ningxia, Hainan, and Taiwan in recent decades. The aim of this study is to clarify the impact of environmental temperature changes on the occurrence and reproduction of *Atractomorpha lata*, and to predict the population’s dynamic changes and occurrence patterns in response to unfavorable temperatures. To establish a theoretical foundation for the comprehensive prevention and control of this pest, it is imperative to enhance fundamental biological research conducted in diverse environmental conditions. Additionally, there is an urgent need to establish an effective prediction and forecasting system. In this experiment, different temperatures were used to observe the developmental period of each insect stage as well as the number of eggs laid by female adults under constant temperature conditions. The developmental threshold temperature and the effective accumulated temperature were then calculated.

## 2. Materials and Methods

### 2.1. Rearing of Insect 

The population of *A. lata* adults were taken from the field of Qujing Academy of Agricultural Sciences (25°17′20.74″ N, 103°48′52.02″ E, and altitude of 1878 m) during July 2022. The experimental population was established in the Laboratory of Insect Biology, School of Biological Resources and Food Engineering, Qujing Normal University, where the host was fed with fresh bean leaves. Before the experiment, a field collection of *A. lata* was placed in an indoor insect feeding cage (50 cm × 50 cm) for three generations. The feeding conditions were as follows: temperature 25 ± 1 °C, relative humidity 70 ± 5%, light condition 16 L:8 D. The average population density was 200 individuals per cage.

### 2.2. Experimental Setup

The five different chambers that can control climate were taken from GXZ380B, Ningbo Jiangnan Instrument Factory, Ningbo, China. The condition of five constant temperatures was 16 °C, 20 °C, 24 °C, 28 °C, and 32 °C. Each chamber had 70% relative humidity and a 16:8 h light–dark cycle. In order to create a breeding environment, sterile sandy soil and nutrient-rich soil were combined in a ratio of 5:1 and placed at the base of the insect container, which had dimensions of 10 cm in diameter and 15 cm in height. Afterwards, a small hole (1 cm diameter) was made into the exterior of every container and covered with a cotton mesh to allow air circulation. The males and females adults, who were in good health and 7 days old, were paired and then moved to the insect canister (100 individuals per temperature treatment). After being given fresh bean leaves to eat, they were placed in a climate chamber. The oviposition times of adult females were regularly recorded (8:00, 20:00), and observations were carried out from egg hatching to nymph development. For each temperature exposure, the first 100 eggs laid were used. Less than 24 h old, newly hatched nymphs were placed into an individual 50 cm centrifuge tube. In order to provide adequate ventilation and individual feeding, every centrifuge tube was punctured with 80 holes (1 mm diameter) in its inner wall. The period of each nymphal instar and survival rates were recorded daily at 8:00 and 20:00.

After emerging all the insects, both male and female adults that emerged on the same day of all 5 chambers were transferred to a new canister. To feed these insects in a new canister, the leaves of soybeans were provided daily. The number of eggs was recorded every day. During this period, if a male was found dead, it was replaced. Simultaneously, the longevity of all adults and female fecundity were monitored daily at 8:00 and 20:00 until the study endpoint, which occurred after all adults had died.

### 2.3. Assessment of Developmental Threshold Temperature and Effective Accumulated Temperature

Using the approaches suggested by Li and Wang [20], both the developmental threshold temperature (*C*) and the effective accumulated temperature (*K*) were assessed at each developmental stage of this insect by the following equations:

(1) $$C=\frac{\sum_{i=1}^{n}T_{i}D_{i}^{2}-\bar{D}\sum_{i=1}^{n}T_{i}D_{i}}{\sum_{i=1}^{n}D_{i}^{2}-n{\bar{D}}^{2}}$$

(2) $$K=\frac{1}{n}\sum_{i=1}^{1}K_{i}$$

where *T_i_* = exposure temperature; *D_i_* = developmental period at *T_i_*; *K_i_* = effective accumulative temperature at the developmental threshold temperature.

### 2.4. Population Trend Index (l) Assessment

By using the data of the life table, the population trend index (*I*) was determined [21].

Population trend index (*I*) = N_1_/N_0_ (3)

N_1_ means the number of eggs after one generation; N_0_ means the number of eggs at contemporary initiation.

If the value of population trend index *I* is greater than 1, it indicates that the next generation is larger than the preceding one; if the value of population trend index *I* is less than 1, it indicates that the next generation is smaller than the preceding one; and if the values equals one, it indicates that the population is developing in a balanced manner.

Number of eggs predicted in the next generation = Adult × Female ratio × Number of eggs laid per female.(4)

### 2.5. Statistical Analysis

The TWOSEX-MSChart application was used to evaluate all of the acquired *A. lata* life table data [22]. Since bootstrap analysis involves random resampling, it is important to minimize the error values. In order to evaluate the variances and standard errors, a paired bootstrap test was used with 100,000 runs [23]. Graphs were constructed using SigmaPlot (version 12.0) software.

While determining the developmental period of each stage of the insects, such as their survival rate and female fertility, we used only the hatching eggs [24]. The population parameters were derived using the equations provided by Chi and Liu [25] and Chi [26] as follows:

(5) $$l_{x}=\sum_{j=1}^{\beta }s_{xj}$$

where *β* is the number of stages, and

(6) $$\frac{\sum_{j=1}^{\beta }s_{xj}s_{xj}}{\sum_{j=1}^{\beta }s_{xj}}$$

Using the Euler–Lotka equation and the iterative bisection approach, the intrinsic rate of increase (*r*) was determined [27]:

(7) $$\sum_{x=0}^{\infty }e^{-r(x+1)}l_{x}m_{x}=1$$

The net reproductive rate (*R*_0_) refers to the cumulative number of offspring that an individual can produce during its lifetime [27]:

(8) $$R_{0}=\sum_{x=0}^{\infty }l_{x}m_{x}$$

The original population at each time period is multiplied by the finite rate of increase (λ), which is then computed as [27] follows:

*λ* = *e*^*r*^ (9)

The mean generation time (*t*) is the period a population needs to increase to *R*_0_-fold of its initial size, given that the population has achieved a stable age-stage distribution [27]. It was calculated using the following method:

(10) $$\frac{\ln(R_{0})}{r}$$

The age-stage life expectancy (*e_xj_*) was determined by the method of Chi and Su [28] as follows:

(11) $$e_{xj}=\sum_{i=x}^{\infty }\sum_{j=y}^{\beta }{S^{\prime }}_{iy}$$

The age-stage reproductive value (*v_xj_*) was calculated by the method of Hu et al. [27] as follows:

(12) $$\upsilon_{xj}=\frac{e^{r\left(x+1\right)}}{S_{xj}}\sum_{i=x}^{\infty }e^{-r\left(i+1\right)}\sum_{y=j}^{\beta }{S^{\prime }}_{iy}f_{iy}$$

## 3. Results

### 3.1. Developmental Period, Longevity, and Fecundity of A. lata on Different Temperature

The developmental period results of developmental stages of *A. lata* are presented in Table 1. As temperature increased, the paired bootstrap test found a significant decrease (*p* < 0.05) in the developmental periods of both egg and preadult development period *A. lata*, indicating that temperature variations influenced its developmental stages. The longest total preadult time was 164.52 ± 0.94 days at 16 °C, while the shortest was 43.21 ± 0.39 days at 32 °C. Total preoviposition period and adult preoviposition period were longest at 16 °C, at 206.28 ± 0.67 days and 37.12 ± 0.63 days, respectively. In contrast, they were shortest at 32 °C, at 52.40 ± 0.81 days and 7.20 ± 0.49 days (Table 1).

The developmental period of each stage was significantly shortened with the increase in temperature. The developmental period was the shortest at 32 °C, and the egg stage was 17.00 ± 0.00 days. The developmental period of 1~5 instar nymphs was (5.29 ± 0.08 d), (4.35 ± 0.10 d), (5.39 ± 0.10 d), (4.94 ± 0.15 d), and (6.21 ± 0.29 d), respectively. The adult longevity decreased significantly with the increase in temperature, and it was significantly higher at 16 °C than at other temperatures. The longevity of female and male adults was (83.40 ± 1.82 d) and (73.59 ± 1.60 d), respectively. As presented in Table 1, the fecundity of *A. lata* was highest at 24 °C (169.56 ± 9.93 eggs), followed by 28 °C (111.88 ± 6.57 eggs).

### 3.2. The Values of Developmental Threshold Temperature and Effective Accumulated Temperature

Table 2 presents of the values of temperature of both parameters of *A. lata* at various developmental stages. The egg, nymph, male adult, and female adult stages had the developmental threshold temperatures of 10.47 °C, 11.06 °C, 13.01 °C, and 11.56 °C, respectively. The values of developmental threshold temperature were lowest at the egg stage (10.47 °C) and highest at the male adult stage (13.01 °C). As shown in Table 2, the values of *C* and *K* for *A. lata* to complete one generation were 11.20 °C and 1356.97 degree-days, respectively.

### 3.3. Age-Stage-Specific Survival Rate

**Figure 1:**
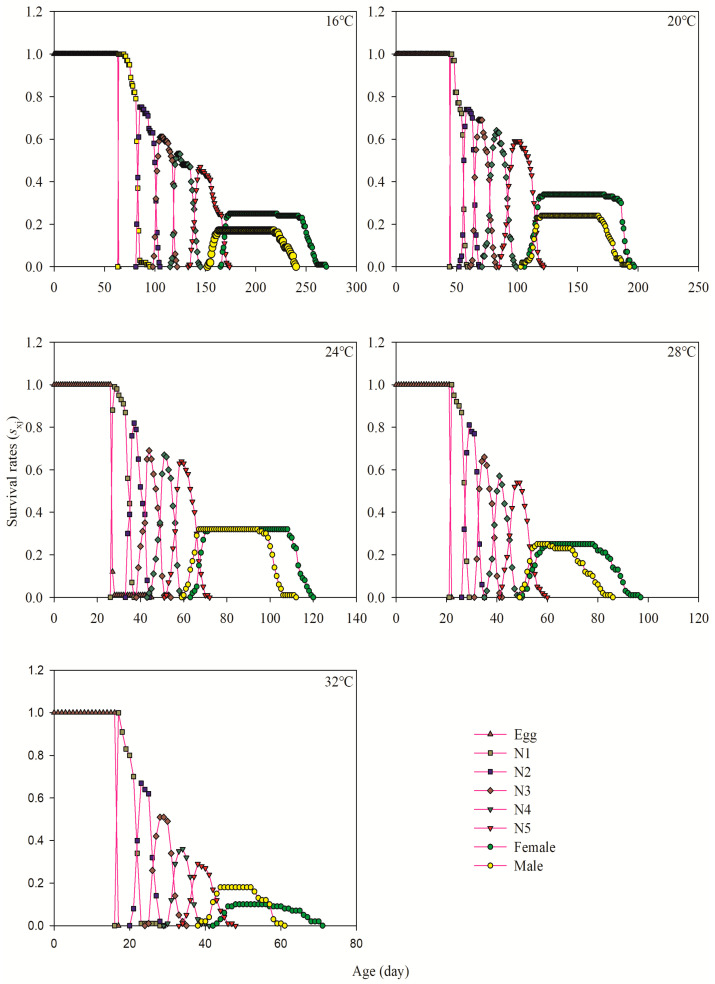
Age-stage-specific survival rates (*s_xj_*) of *A. lata* at different temperatures. Note: N1–N5 represent the first, second, third, fourth, and fifth nymph stages, respectively.

Results showed that *A. lata* was able to complete all its stages from eggs to adult at all temperatures. No clear overlap was found in the age-stage–survival curves, which considers the individual differences in developmental rates under different temperatures. The hatching rate of eggs was more than 90% at 20~28 °C. The highest and lowest survival rates of female adults were 20 °C (34%) and 32 °C (10%), respectively, while the highest and lowest survival rates of adult male were 24 °C (32%) and 16 °C (17%), respectively (Figure 1).

### 3.4. Age-Specific Survivability and Age-Stage-Specific Fecundity

**Figure 2:**
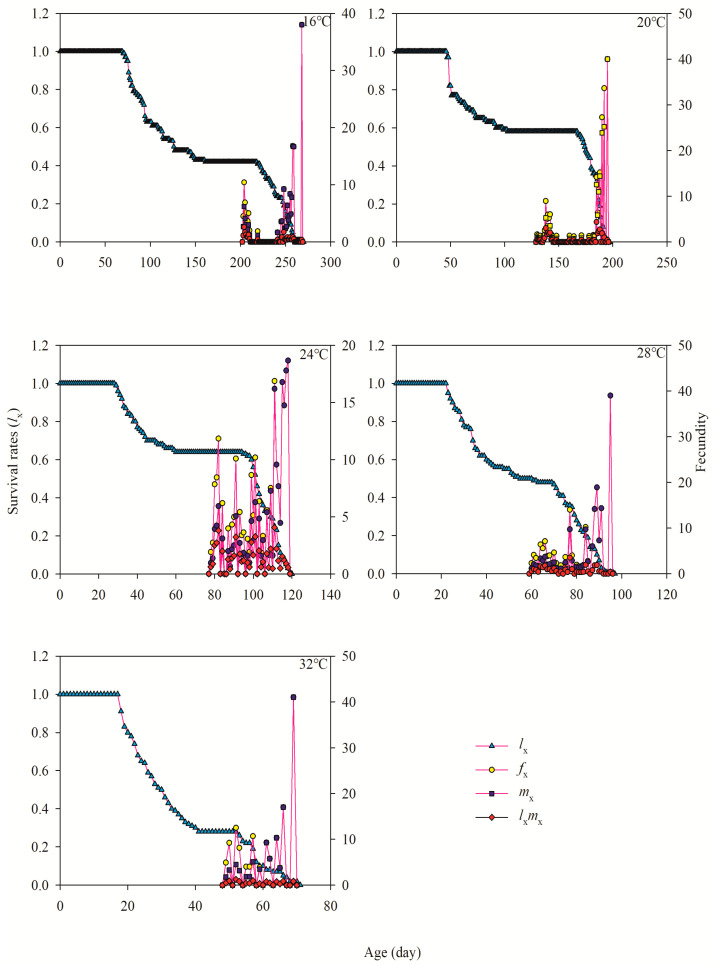
Age-specific survival (*l_x_*), age-stage-specific fecundity (*f_x_*), age-specific fecundity (*m_x_*), and age-specific maternity (*l_x_m_x_*) of *A. lata* at different temperatures.

The age-stage-specific fecundity (*f_x_*), age-specific survival rate (*l_x_*), age-specific maternity (*l_x_m_x_*), and fecundity of the total population (*m_x_*) of *A. lata* regimes are demonstrated in Figure 2. The age-specific survival rates (*l_x_*) exhibited a significant decline as the age of test insects increased across the range of temperatures tested, reaching from 16 to 32 °C (Figure 2). The stoppage of oviposition(*m_x_*) occurred at ages 269.00, 196.00, 118.00, 96.00, and 69.00 days at temperatures of 16 °C, 20 °C, 24 °C, 28 °C, and 32 °C, respectively. The values of *l_x_* exhibited a gradual decrease with increasing age at 16 °C but a sharp decline at 32 °C (Figure 2). The curves representing the daily egg-laying rate per female (*f_x_*) and (*m_x_*) reached their highest point at a temperature of 32 °C (Figure 2).

### 3.5. Age-Stage-Specific Life Expectancy

**Figure 3:**
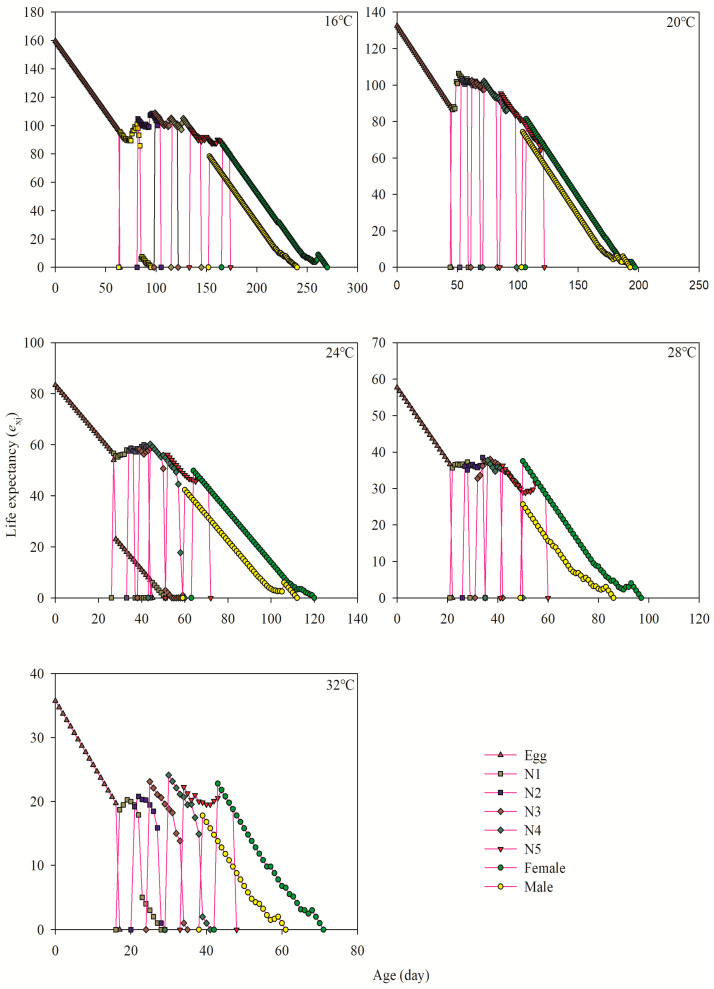
Life expectancy (*e_xj_*) of each age-stage group of *A. lata* at different temperatures. N1–N5 represent the first, second, third, fourth, and fifth nymph stages, respectively.

The life expectancy (*e_xj_*) value of the same stage decreased as the temperature increased, while the *e_xj_* value of the same stage gradually declined as the age increased. According to the data presented in Figure 3, the maximum life expectancy for adult females was determined at a temperature of 16 °C, with a value of 86.56 days. Conversely, the minimum life expectancy was recorded at a temperature of 32 °C, with a value of 22.80 days. Similarly, male adults showed the highest average life expectancy at 16 °C (78.29 days), whereas the shortest life expectancy was observed at 32 °C (17.78 days, Figure 3).

### 3.6. Age-Stage-Specific Reproductive Value

**Figure 4:**
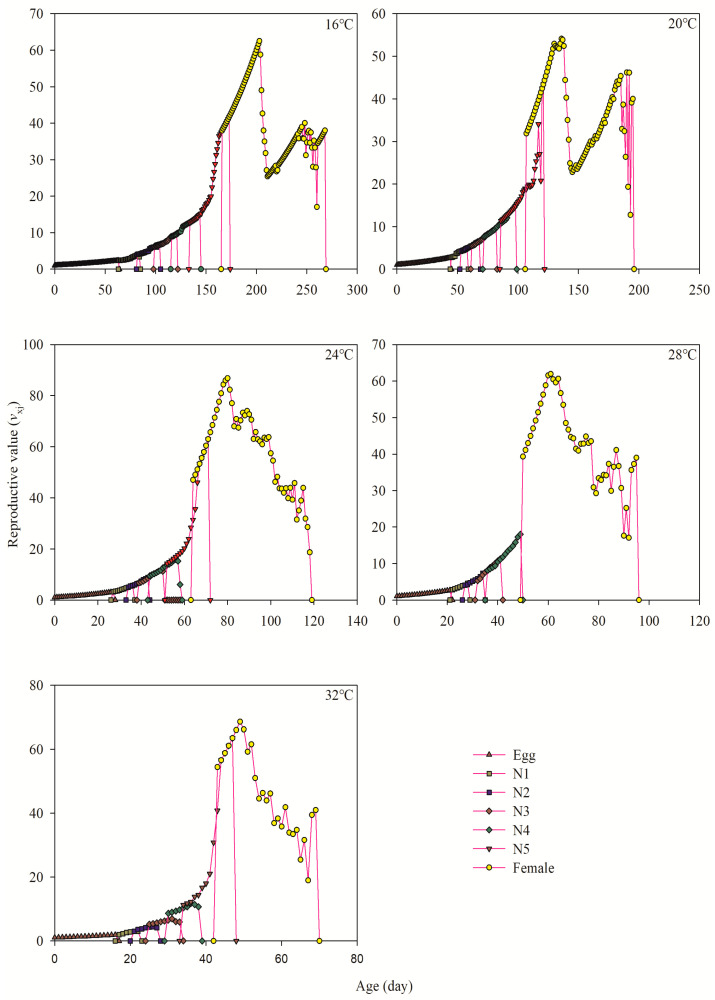
Reproductive value (*v_xj_*) of each age-stage group of *A. lata* at different temperatures. N1–N5 represent the first, second, third, fourth, and fifth instar, respectively.

The values of *v_xj_* exhibited a progressive increase as age (*x*) and stage (*j*) increased, as indicated in Figure 4. Within the temperature range of 16 °C to 32 °C, the first age-stage *v_xj_* values of female adults were observed at 166.00 d, 107.00 d, 64.00 d, 50.00 d, and 43.00 d, respectively. Furthermore, the highest *v_xj_* values for adult females were recorded at ages 203.00 d (62.53 d^−1^), 136.00 d (54.14 d^−1^), 80.00 d (86.83 d^−1^), 61.00 d (62.00 d^−1^), and 49.00 d (68.60 d^−1^), respectively, as shown in Figure 4.

### 3.7. Population Parameters

**Figure 5:**
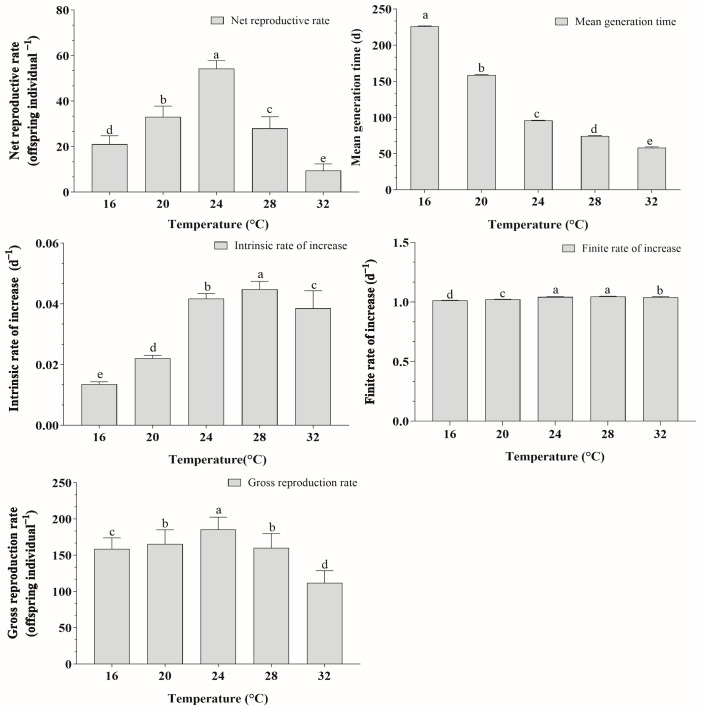
Population parameters of *Atractomorpha lata* at various temperatures. Data are presented as mean ± SE. Histograms with different letters indicate significant differences based on the paired bootstrap test at *p* < 0.05. Standard errors were estimated using 100,000 bootstrap resampling iterations.

The parameters for *A. lata* population are listed in Figure 5. The mean generation time (*T*) was longest at 16 °C (226.00 ± 0.70 days) and shortest at 32 °C (58.26 ± 1.05 days). The intrinsic growth rate initially increased and then decreased with rising temperature, peaking at 28 °C. Within a certain range, an increase in temperature can boost the population growth rate, but excessively high temperatures are detrimental to population growth. Between 24 °C and 28 °C, there was no significant difference in the finite rate of increase (*λ*) of the population. The highest net reproductive rate (*R*_0_) and total reproductive rate (*GRR*) were observed at 24 °C (54.26 ± 8.49 offspring and 185.53 ± 16.94 offspring, respectively), while the lowest were at 32 °C (9.43 ± 2.91 offspring and 111.80 ± 16.80 offspring, respectively).

### 3.8. Population Trend Index Response to Temperature Gradient

The life table for the experimental population was established using the observed data on survival rate, female ratio, and egg production per female. Table 3 illustrates that the initial egg count was based on 100 eggs. The population trend index (*I*) values were all greater than 1, suggesting a sustainable growth trend under the experimental temperature conditions. The highest *I* value, 61.64, was recorded at 24 °C, followed by 12.34 at 32 °C.

## 4. Discussion

The climate pattern determines insect population distribution, growth, and development [19]. Grasshoppers are widely distributed and harmful pest groups in agricultural and animal husbandry production. They are distributed on all continents except Antarctica. Climate warming increases the overwintering survival rate of locusts, accelerates their development rate, increases the number of insect sources, advances the occurrence period of locust nymphs, and prolongs the damage time [19,29,30].

The change in environmental temperature directly affects the development, reproduction, and regional distribution of grasshopper population. In the process of its growth and development, the effective accumulated temperature required to complete a certain development stage is fixed, so its development period will change with the change of temperature [6]. Many studies have reported the effect of temperature on physiological and behavioral characteristics of different grasshoppers; some of them are *Calliptamus italicus*, *Gomphocerus sibiricus*, *Chorthippus albonemus*, etc. [31,32,33]. For example, Wei et al. [34] studied the adaptability of *Calliptamus abbreviatus* to temperature and found that the developmental period of 1st~5th instar nymphs at 18 °C was 2.91~4.59 times that at 33 °C. Similar results were obtained in current experiment. The inverse relation was found between the period of developmental stage and the increase in temperature within the range limit of 16 °C to 32 °C *A. lata*. The period of developmental stages were found to be considerably reduced at 32 °C compared to 16 °C. The results are similar to the above locust research findings, where consistency was found between the ambient temperature and development of thermophilic animals [6]. The specific age–instar survival rate (*S_xj_*) curve showed that the harmful locust could survive in the experimental temperature range, and the egg’s hatching rate and larvae were higher at 20~28 °C. However, the survival rate of adult stages of both sexes significantly decreased at 32 °C, which proved that high temperatures greatly influence the population survival rate, and temperatures above 28 °C are not suitable for its growth and development. The survival rate (*S_xj_*) curve for a specific age instar showed that the harmful locust could survive within the experimental temperature range. The hatching rate of eggs and the survival rate of larvae were higher at 20~28 °C. In this study, under the test conditions, the adult preoviposition period (APOP) was excluded from the preadult stage, thereby ignoring the contribution of reproductive age in regulating the insect population rate. Meanwhile, the total preoviposition period (TPOP) was used to accurately describe the contribution of the initial reproductive age to the intrinsic growth rate. Gabre et al. [35] found that the peak *v_xj_* value of female adults of *Chrysomya megacephala* (Fabricius) occurred after the initial reproductive age and was comparable to its total preoviposition periods value. In this work, the maximal reproduction values of adult female after emergence were recorded at 203.00 d (62.53 d^−1^), 136.00 d (54.14 d^−1^), 80.00 d (86.83 d^−1^), 61.00 d (61.94 d^−1^), and 49.00 d (68.60 d^−1^) (Figure 4), while the TPOP values were at 206.28 ± 0.67, 141.91 ± 2.14, 83.19 ± 0.67, 66.00 ± 0.83, and 52.40 ± 0.81 d (Table 1), respectively. 

The strength of female reproductive ability is a key factor in evaluating insect population growth and achieving generational continuity, and its strength is also regulated by environmental temperature. When exposed to either low or high temperatures, the development of female sexual glands can be disrupted, leading to inhibited egg maturation and a reduced number of eggs laid [11]. The results of this study indicate that the reproductive capacity of female adults initially increases and then decreases with rising temperatures. The highest average egg laying per female occurs at 24 °C (169.56 ± 9.93 eggs), while the lowest is at 16 °C (84.12 ± 2.26 eggs), indicating that low temperatures are not conducive to egg laying. The average summer temperature in Qujing typically ranges between 24 and 26 °C. Based on the field living habits of the long-tailed locusts, such as hiding under host leaves during noon temperature spikes, it is inferred that under suitable environmental conditions, outbreaks of the long-tailed locust may occur. However, if the long-tailed locusts do not reach a certain physiological age, the mortality rate remains stable. This can result in prolonged damage to crops, significantly impacting crop growth and leading to economic losses. Based on the generation cycle, male–female insect ratio, and egg production of long-headed harmful locusts under low-temperature conditions, it is speculated that unfavorable external environments will lead to an increase in the number of female insects emerging, and the population will produce more offspring to compensate for the impact of unfavorable environments on individual population numbers. Meanwhile, we found that *A. lata* has ability to complete its generations normally within the range of 16 to 32 °C, and at 24 °C, optimal reproductive growth was found; these results are similar to previous findings about *Atractomorpha sinensis* [6].

The distribution of the pest’s geography and altitude can be determined using the starting point temperature for development and the effective accumulated temperature. By combining these with local temperatures, the theoretical occurrence of the pest in the local area can be inferred, allowing us to determine the timing and frequency of pest control [36]. According to the law of effective accumulated temperatures, the different generations of the harmful locust across different geographical latitudes can be explained, and the development period of different insect stages can be inferred [6]. For instance, *A. sinensis* can have 2~3 generations per year in Jiangxi Province (34°29′), while only one generation occurs per year in Shanxi and Shandong Provinces (34°22′~36°25′) [37]. According to the results of this study, the developmental threshold temperature (10.47 °C) of the eggs was the lowest (10.47 °C), indicating that the eggs had a relatively strong ability to adapt to low temperatures, which was similar to the developmental threshold temperature of overwintering eggs of other harmful locust species in the same latitude area. This may be related to the overwintering of harmful locusts in the soil as overwintering eggs [38]. It is speculated that the insect can better adapt to a low-temperature environment in high-altitude areas [10,37]. The annual effective accumulated temperature in Yunnan Province is 3500~4200 °C. According to the calculation formula M = *K*_1_/*K* (M represents the number of generations per year, *K*_1_ represents the annual effective total accumulated temperature in a certain place, and *K* represents the effective accumulated temperature required for a certain insect species to complete a generation), the theoretical number of long-term harmful locust generations in Yunnan Province is calculated to be 2~3; during the field survey, similar results were found [36,39]. Li et al. [7] calculated the developmental threshold temperature for eggs in the Changchun (43°05′ N) population to be 10.81 °C using linear regression, a result similar to that of this study. The latitude span for the Li population in the Qujing and Changchun areas is quite broad. The development threshold temperature results were similar, even though the estimation methods and host plants were different. This is because the test insects’ natural habitat is in a subtropical plateau monsoon climate area with low latitude and high elevation. The annual average temperature is about 22 °C, the spring temperature is unstable, the summer is not hot, and the winter is mild [10]. It is classified as a natural “thermostat”. In this area, the harmful locust is sensitive to the environment’s high-temperature response. The negative locust population is prone to the potential threat of a field outbreak at the optimum temperature of 24 °C.

Predicting the nonoccurrence dynamics of pest populations and formulating scientific prevention and control premises require accurate calculation of their population ecological parameters [13]. Establishing a life table is crucial for studying the population ecology of pests and formulating pest control strategies under varying environmental conditions. This method helps in analyzing population dynamics, predicting future growth trends, and determining optimal control timings [40]. To study the influence of environmental factors on the population dynamics and management, the life table is regarded as an important tool [26,41]. Parameters such as the population trend index (*I*), net reproduction rate (R0), and intrinsic rate of increase (*r*) play significant roles in describing insect population dynamics, including growth, survival, and reproduction rates [27,40,42]. In our experiment at the temperature gradient between 16 and 32 °C, the population trend index values were more than 1, which indicates that the subsequent population is larger than the previous population. The higher values of this index were observed at 24 °C, suggesting rapid population growth. In addition, both the net reproduction rate (*R*_0_) and gross reproductive rate (*GRR*) peaked at 24 °C, implying strong fertility performance at this temperature. Notably, *A. lata* exhibited the lowest fecundity at 16 °C. Nevertheless, at 32 °C, low oviposition rates result in a poor net reproductive rate. Over the past three years, Qujing has experienced an increase in annual extreme daily mean temperatures [43], consistent with the significant rise in global mean surface air temperature [44]. Given the potential for even higher temperatures in the future, our research suggests that the *A. lata* population may decline and degenerate when the mean temperature reaches 32 °C. Compared to less-disturbed habitats with lower historical climate warming rates, intensive agricultural land use and historical climate warming are linked to nearly 50% declines in insect abundance and 27% in species number within insect assemblages [45]. According to Chi [26], *R*_0_ = *F* × (female %); this result is consistent with Chi [26]. Although the highest values of *r* and *λ* were recorded at 28 °C, the net growth rate was lower than at 24 °C. Additionally, the survival rate of adults, the female ratio, and the number of eggs laid per female were lower at 28 °C compared to other temperatures, which could potentially result in slower population growth. Therefore, a single life parameter cannot accurately measure the developmental potential of the population, and it is necessary to comprehensively analyze the parameters of the population life table. According to our research findings, 24 °C is the ideal temperature for *A. lata* survival and reproduction. These population parameter patterns are in accordance with findings for other insects. A similar effect caused by the values of *I* and fecundity has also been reported in *A. lata* [7].

## 5. Conclusions

Through the construction and analysis of the life table of the harmful locust population, we found that 24 °C is the optimal ambient temperature for the growth, development, and reproduction of the harmful locust population in Qujing. Both high and low temperatures are not conducive to population growth and development. However, the dynamics and growth and development of insect populations are not only affected by environmental temperature disturbances, such as hosts, pesticides, and precipitation [46,47,48]. Therefore, due to the interaction among various factors, there may be differences between observing the population dynamics of *A. lata* under stable indoor conditions and its dynamics in a natural environment. Therefore, it is necessary to combine these factors for further research to more systematically and comprehensively understand the occurrence dynamics and spatial distribution of the *A. lata* population.

## Figures and Tables

**Table 1:** Response of different developmental stages of *Atractomorpha lata* to different temperatures.

Developmental Stage	Developmental Period at Different Temperatures									
	16 °C		20 °C		24 °C		28 °C		32 °C	
	*n*	Days	*n*	Days	*n*	Days	*n*	Days	*n*	Days
Egg	100	64.00 ± 0.00 a	100	45.00 ± 0.00 b	100	27.29 ± 0.18 c	100	22.00 ± 0.00 d	100	17.00 ± 0.00 e
1st instar	75	19.36 ± 0.12 a	77	11.19 ± 0.13 b	84	8.13 ± 0.12 c	85	5.82 ± 0.07 d	69	5.29 ± 0.08 d
2nd instar	61	18.28 ± 0.17 a	71	9.23 ± 0.12 b	75	6.41 ± 0.10 c	76	5.34 ± 0.06 d	52	4.35 ± 0.10 e
3rd instar	54	17.46 ± 0.10 a	65	12.25 ± 0.23 b	68	6.87 ± 0.08 c	62	5.90 ± 0.11 d	38	5.39 ± 0.10 d
4th instar	48	20.42 ± 0.39 a	60	14.75 ± 0.10 b	64	7.25 ± 0.09 c	56	6.11 ± 0.14 d	32	4.94 ± 0.15 e
5th instar	42	24.69 ± 0.66 a	58	21.66 ± 0.33 b	64	9.89 ± 0.23 c	50	8.32 ± 0.24 d	28	6.21 ± 0.29 e
Total Preadult stage	42	164.52 ± 0.94 a	58	114.00 ± 0.48 b	64	65.97 ± 0.32 c	50	53.58 ± 0.34 d	28	43.21 ± 0.39 e
Female adult	25	83.40 ± 1.82 a	34	74.18 ± 0.84 b	32	46.09 ± 0.41 c	25	32.92 ± 0.80 d	10	20.60 ± 1.18 e
Male Adult	17	73.59 ± 1.60 a	24	64.67 ± 1.25 b	32	38.16 ± 0.48 c	25	23.08 ± 1.20 d	18	14.67 ± 0.57 e
APOP	25	37.12 ± 0.63 a	34	27.65 ± 2.04 b	32	15.41 ± 0.58 c	25	11.40 ± 0.54 d	10	7.20 ± 0.49 e
TPOP	25	206.28 ± 0.67 a	34	141.91 ± 2.14 b	32	83.19 ± 0.67 c	25	66.00 ± 0.83 d	10	52.40 ± 0.81 e
Fecundity (F) (eggs laid/female)	25	84.12 ± 2.26 d	34	97.15 ± 3.03 c	32	169.56 ± 9.93 a	25	111.88 ± 6.57 b	10	94.30 ± 7.78 c

Note: APOP, adult preoviposition period; TPOP, total preoviposition period. Data are presented as mean ± SE. The terms “Male adults” and “Female adults” represent the lifespan of male and female insects, respectively. Different letters in the same row indicate significant differences based on the paired bootstrap test at *p* < 0.05. Standard errors were estimated by 100,000 bootstrap resamples.

**Table 2:** The values of temperatures of developmental threshold effective accumulative for the different growth stages of *Atractomorpha lata*.

Developmental Stage	Developmental Threshold Temperature (°C)	Effective Accumulative Temperature (Degree-Days)	Coefficient of Variation (cv)
Egg	10.47	380.89	0.077
1st instar	10.74	102.86	0.049
2nd instar	11.13	86.84	0.050
3rd instar	10.07	108.97	0.099
4th instar	11.76	99.26	0.140
5th instar	12.25	126.01	0.217
Nymph	11.06	534.52	0.096
Preoviposition	12.31	170.10	0.183
Female adult	11.56	506.39	0.211
Male adult	13.01	343.24	0.280
Generation	11.20	1356.97	0.126

**Table 3:** Gradient temperature effects on the developmental stages of *Atractomorpha lata*.

Developmental Stage	Number of Individuals Entering Various Developmental Stages				
	16 °C	20 °C	24 °C	28 °C	32 °C
Eggs	100.00	100.00	100.00	100.00	100.00
1st instar	87.41	95.62	96.57	91.48	89.55
2nd instar	71.10	88.17	85.21	81.79	67.49
3rd instar	62.94	80.72	77.26	66.73	49.32
4th instar	55.94	74.51	72.71	60.27	41.53
5th instar	48.95	72.03	72.71	53.81	36.34
Adult	48.95	72.03	72.71	53.81	36.34
Ratio of female	0.59	0.59	0.50	0.50	0.36
Eggs laid/female	84.12	97.15	169.56	111.88	94.30
Number of eggs expected in the following generation	2429.48	4128.39	6164.48	3010.23	1233.64
Population trend index (*I*)	24.29	41.28	61.64	30.10	12.34

## Data Availability

The data presented in this study are available on request from the corresponding author.
